# Synthesis and Electronic Properties of Novel Donor–π–Acceptor-Type Functional Dyes with a Carbonyl-Bridged Bithiophene π-Spacer

**DOI:** 10.3390/molecules30153084

**Published:** 2025-07-23

**Authors:** Miyu Ueda, Ryo Nagayama, Masaki Nagaoka, Naoya Suzuki, Shintaro Kodama, Takeshi Maeda, Shin-ichiro Kato, Shigeyuki Yagi

**Affiliations:** Department of Applied Chemistry, Graduate School of Engineering, Osaka Metropolitan University, 1-1 Gakuen-cho, Naka-ku, Sakai 599-8531, Osaka, Japan

**Keywords:** donor–π–acceptor-type dye, intramolecular charge transfer, panchromatic absorption, photostability, frontier orbital

## Abstract

In this study, we synthesized novel donor–π–acceptor (D–π–A) functional dyes bearing a carbonyl-bridged bithiophene as a π-conjugated spacer and evaluated the absorption and fluorescence properties as well as the photostability. The developed dyes **1-CO**–**3-CO** possess an *N*,*N*-diphenylaminophenyl electron donor unit and an electron acceptor unit such as a formyl group (**1-CO**), an (*N*,*N*-diethylthiobarbituryl)methylene moiety (**2-CO**), or a (3-dicyanomethylidene-1-indanon-2-yl)methylene moiety (**3-CO**). The absorption spectra of **1-CO**–**3-CO** in dichloromethane at room temperature showed absorption maxima at 569 nm, 631 nm, and 667 nm, respectively, and the stronger acceptors in **2-CO** and **3-CO** led to enhancement of the ICT character. In addition, **2-CO** and **3-CO** had a second absorption band in the visible region, showing panchromatic absorption properties. Electrochemical analyses of the developed dyes revealed that the carbonyl bridging group in the π-spacer contributes to stabilization of the frontier orbitals such as the highest occupied and lowest unoccupied molecular orbitals (HOMO and LUMO, respectively), in comparison with the referential dyes bearing a dibutylmethylene-bridged bithiophene spacer, **1-CBu_2_**–**3-CBu_2_**. The HOMO/LUMO stabilization brought about high photostability in the doped poly(methyl methacrylate) film.

## 1. Introduction

Donor–π–acceptor (D–π–A) functional dyes consist of electron-donating and electron-withdrawing groups linked by a π-conjugated spacer and usually exhibit intense absorption in the visible region due to the intramolecular charge transfer (ICT) transition from the donor unit to the acceptor one [1,2,3]. These dyes also have large dipole moments in the excited state, and thus the large Stokes shifts caused by solvent reorientation and structural relaxation often result in suppression of self-quenching of fluorescent emission. The optical and/or photophysical properties of D–π–A dyes are strategically tunable because the main electronic transition is based on the ICT from the highest occupied molecular orbital (HOMO) localized around the donor to the lowest unoccupied molecular orbital (LUMO) localized around the acceptor: optimizing the electron-donating and electron-withdrawing properties of the donor and the acceptor, respectively, as well as tuning the effective π-conjugation length allows us to obtain rationally designed D–π–A dyes according to the purposes [1,4]. Thus, a wide range of D–π–A dyes have so far been utilized in various fields such as photosensitizers for dye-sensitized solar cells (DSSCs) [5,6,7,8,9,10,11,12], fluorescent emitters for organic light-emitting diodes [13,14,15,16], non-linear optical materials [17,18,19,20,21], and fluorescent probes for bio-imaging [22,23,24,25,26,27].

Taking practical applications of D–π–A dyes into consideration, excellent photostability is highly required in addition to favorable spectroscopic properties [28,29,30]. The donor and acceptor units in a D–π–A system are often connected to each other by a C=C bond-based π-conjugation bridge. However, such a structure readily undergoes *trans*–*cis* photoisomerization, which may reduce the photostability [31,32,33]. Furthermore, extending the D–π–A π-conjugation system increases electron richness, leading to high photo-reactivity [34].

**Figure 1:**
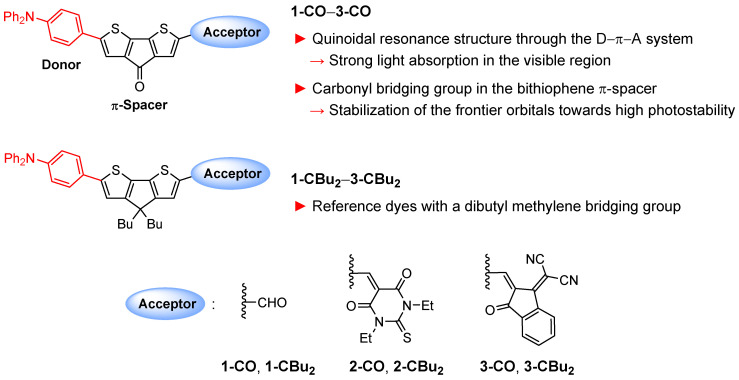
Molecular design of D–π–A-type functional dyes in this work.

In this study, we have aimed to design and synthesize D–π–A dyes with high photostability by employing a carbonyl-bridged bithiophene spacer (Figure 1). The dyes **1-CO**–**3-CO** consist of a diphenylaminophenyl group as a donor and a series of acceptors such as a formyl group (**1-CO**), an (*N*,*N*-diethylthiobarbituryl)methylene moiety (**2-CO**), and a (3-dicyanomethylidene-1-indanon-2-yl)methylene moiety (**3-CO**) linked by a carbonyl-bridged bithiophene spacer. The introduction of bithiophene is expected to contribute to the extension of π-conjugation through a quinoidal resonance structure and promote strong absorption in the visible region. Furthermore, the electron-withdrawing carbonyl group at the bridging position of the bithiophene skeleton is expected to lower the energy levels of the frontier orbitals to lead to high photostability. To investigate the effect of the carbonyl bridging group on the spectral properties and photostability, we also prepare the reference dyes with a dibutylmethylene-bridged bithiophene spacer, namely **1-CBu_2_**–**3-CBu_2_**. Here, we report the electronic properties of the developed D–π–A dyes and discuss how the frontier orbitals are turned by the bridged bithiophene spacer and the acceptor group. Also, we demonstrate how effectively the carbonyl-bridged bithiophene spacer improves the photostability of the present D–π–A chromophoric system.

## 2. Results and Discussion

### 2.1. Synthesis and Characterization

**Scheme 1:**
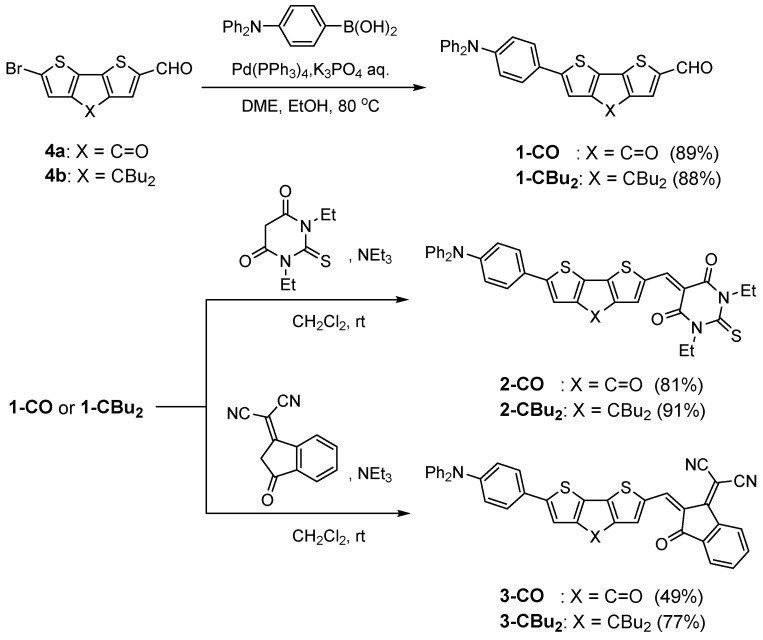
Synthesis of **1-CO**–**3-CO** and **1-CBu_2_**–**3-CBu_2_**. DME represents 1,2-dimethoxyethane.

The dyes **1-CO**–**3-CO** and **1-CBu_2_**–**3-CBu_2_** were synthesized according to Scheme 1. 6-Bromo-4*H*-cyclopenta[2,1-*b*:3,4-*b′*]dithiophene-4-one-2-carbaldehyde (**4a**) was synthesized according to the reported method [12]. The dye **1-CO** was prepared by the Suzuki–Miyaura cross-coupling reaction of **4a** with 4-(*N*,*N*-diphenylamino)phenylboronic acid. The dyes **2-CO** and **3-CO** were obtained by the Knoevenagel condensation of **1-CO** with 1,3-diethyl-2-thiobarbituric acid and 3-(dicyanomethylidene)indan-1-one, respectively. The dyes **1-CBu_2_**–**3-CBu_2_** were synthesized according to a similar method to the preparation of **1-CO**–**3**-**CO**, where 6-bromo-4,4-dibutyl-4*H*-cyclopenta[2,1-*b*:3,4-*b′*]dithiophene-2-carbaldehyde (**4b**) was prepared according to the reported method [35]. The prepared dyes were characterized by ^1^H NMR, ^13^C NMR, and ESI-TOF mass spectra, as well as elemental analyses.

### 2.2. UV–vis–NIR Absorption Properties

**Figure 2:**
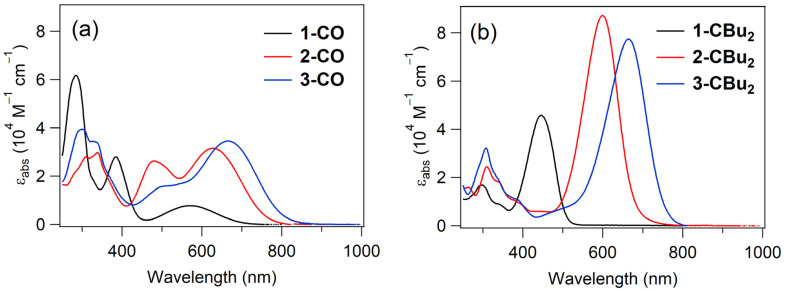
UV–vis–NIR absorption spectra of (**a**) **1-CO**–**3-CO** and (**b**) **1-CBu_2_**–**3-CBu_2_** in dichloromethane at room temperature.

The optical properties of **1-CO**–**3-CO** and **1-CBu_2_**–**3-CBu_2_** were investigated in dichloromethane under ambient conditions. The UV–vis–near-infrared (NIR) absorption spectra of these dyes were shown in Figure 2, and the spectroscopic data were summarized in Table 1. As shown in Figure 2a, **1-CO**–**3-CO** showed the lowest-energy absorption maxima (λ_abs_s) at 569, 631, and 667 nm, respectively, with molar absorption coefficients (ε_abs_s) of 7800–34,600 mol^−1^ L cm^−1^. Due to the π-extension at the acceptor terminal, **2-CO** and **3-CO** exhibited more red-shifted λ_abs_s in comparison with **1-CO**. Notably, **3-CO** showed the most pronounced bathochromic shift among **1-CO**–**3-CO**, indicating that the stronger acceptor in **3-CO** most effectively enhanced the ICT character, as observed in the previous reported merocyanine-type chromophores [1,36,37,38]. Interestingly, **2-CO** and **3-CO** had the second absorption bands in the visible region at 481 (ε_abs_ = 24,300 mol^−1^ L cm^−1^) and 498 nm (ε_abs_ = 15,700 mol^−1^ L cm^−1^), respectively, to show panchromatic absorption properties, although **1-CO** had the second band at 383 nm (ε_abs_ = 27,900 mol^−1^ L cm^−1^) in the near ultraviolet region. On the other hand, as shown in Figure 2b, **1-CBu_2_**–**3-CBu_2_** showed single absorption bands in the visible region, the λ_abs_s of which were observed at 445, 598, and 665 nm, respectively. The values of ε_abs_ were much larger than **1-CO**–**3-CO**, ranging from 45,800 to 87,200 mol^−1^ L cm^−1^. The trend of the red shift in the lowest-energy absorption band among **1-CBu_2_**–**3-CBu_2_** was the same as those among **1-CO**–**3-CO**, indicating that these absorption bands are attributed to the electronic transition based on the ICT from the donor unit to the acceptor one. One can see that the panchromatic absorption spectra of **1-CO**–**3-CO** should be caused by introduction of the carbonyl bridging group to the bithiophene spacer. The effect of this carbonyl bridging group on the optical properties is discussed later from the theoretical study in Section 2.4.

### 2.3. Photoluminescence Properties

**Figure 3:**
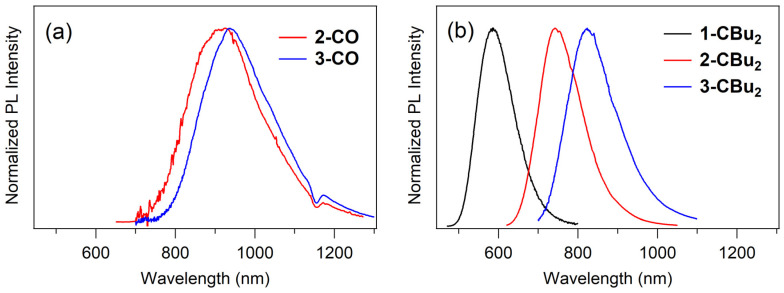
Photoluminescence spectra of (**a**) **2-CO** and **3-CO** and (**b**) **1-CBu_2_**–**3-CBu_2_** in dichloromethane at room temperature.

Photoluminescence (PL) spectra of **2-CO**, **3-CO**, and **1-CBu_2_**–**3-CBu_2_** were obtained in dichloromethane at room temperature, as shown in Figure 3. The PL and photophysical data are also summarized in Table 2. The dye **1-CO** did not exhibit any PL, whereas **2-CO** and **3-CO** did very weak NIR emission with PL maxima (λ_PL_s) at 926 nm for **2-CO** and 937 nm for **3-CO** (*Φ*_PL_s < 0.01). The large Stokes shifts (Δ$\tilde{\nu }$_Stokes_s) of **2-CO** and **3-CO** ($\Delta \tilde{\nu }$_Stokes_ = 5120 and 4320 cm^−1^, respectively) are obviously caused by their remarkable ICT characters. On the other hand, **1-CBu_2_** and **2-CBu_2_** exhibited intense emission with λ_PL_s at 592 and 742 nm (*Φ*_PL_ = 0.93 and 0.48), respectively, accompanied by large $\Delta \tilde{\nu }$_Stokes_s of 5440 and 3190 cm^−1^ for **1-CBu_2_** and **2-CBu_2_**, respectively. The nanosecond-order PL lifetimes (τ_PL_s) and photophysical parameters (radiative and non-radiative rate constants *k*_r_ and *k*_nr_, respectively) clarified the observed emission was exclusively fluorescence. The dye **3-CBu_2_** exhibited NIR emission at 823 nm with a modest *Φ*_PL_ of 0.03, accompanied by a Δ$\tilde{\nu }$_Stokes_ of 2860 cm^−1^. The weak fluorescence of **1-CO**–**3-CO** in comparison with **1-CBu_2_**–**3-CBu_2_** is primarily attributed to the intersystem crossing from the singlet excited state to the triplet state caused by the carbonyl-bridged structure: the significant fluorescent quenching for **1-CO**–**3-CO** should be caused according to the El-Sayed’s rule [39,40], although their phosphorescence spectra were silent in deaerated glassy 2-methyl THF at 77 K. One might also see that negligible or weak PL of **1-CO**–**3-CO** is attributed to a fast non-radiative decay process from the singlet excited state to the ground state. Specifically, NIR emissive dyes **2-CO** and **3-CO** are likely to undergo highly facilitated non-radiative decay according to the energy gap law [41,42]. Of particular note is the high quantum yield of NIR fluorescence from **2-CBu_2_** (*Φ*_PL_ = 0.48), which is comparable to those of representative NIR fluorescent dyes in the same λ_PL_ region [35,43,44,45,46,47].

We also investigated the solvatochromic properties of the present dyes, and the UV–vis–NIR absorption and PL spectra in various solvents are shown in Supplementary Materials (Figures S7 and S8, respectively). Solvent effects are more remarkable for the PL spectra rather than the absorption spectra, although PL spectral changes were not evaluated for the non-luminescent dye **1-CO**. As usually observed for neutral ICT-type dyes, the PL spectra of the present dyes undergo red shifts with increasing solvent polarity, reflecting solvent-induced stabilization of the excited state [48,49,50].

### 2.4. Theoretical Calculations

**Figure 4:**
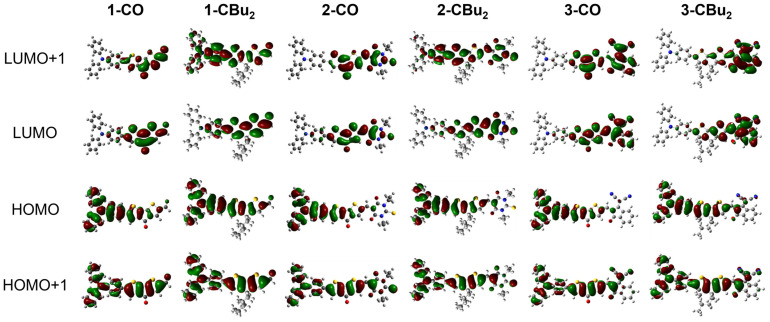
Distributions of frontier orbitals of **1-CO**–**3-CO** and **1-CBu_2_**–**3-CBu_2_**. obtained by DFT and TD-DFT calculations at the M062X/6-31G(d,p) level of theory with the SMD solvation model in dichloromethane.

Optical properties of the D–π–A dyes were further studied by density functional theory (DFT) and time-dependent DFT (TD-DFT) calculations on the Gaussian 09 program package [51]. The ground state (S_0_) structures of all the dyes were optimized at the M062X/6-31G(d,p) level of theory with the SMD solvation model in dichloromethane, and the distributions of the frontier orbitals (HOMO–1, HOMO, LUMO, and LUMO+1) are shown in Figure 4. The HOMO and LUMO energy levels are summarized in Supplementary Materials (Table S1). The contributions of the donor, π-spacer, and acceptor units to the molecular orbitals were calculated by the method in the literature [52], and the data are summarized in Supplementary Materials (Table S2). In the case of **1-CO**–**3-CO**, the HOMO is localized mainly at the *N*,*N*-diphenylaminophenyl donor moiety (76.0–80.5%), and the HOMO–1 is delocalized from the donor (28.8–32.7%) to the carbonyl-bridged bithiophene π-spacer (50.8–63.3%), accompanied by the contribution of the acceptor moiety at some extent (4.0–20.4%). No orbital distribution is found on the carbonyl bridging group. On the other hand, the LUMO is localized from the π-spacer (40.9–89.6%) to the acceptor moiety (6.4–56.9%). The contribution of the acceptor moiety to the LUMO increases in the order of **1-CO** < **2-CO** < **3-CO**, indicating that the stronger acceptor induces more remarkable ICT. The LUMO+1 possesses a similar orbital contour to the LUMO, although the contribution of the carbonyl bridging group in the LUMO+1 is larger than that in the LUMO. In the case of **1-CBu_2_**–**3-CBu_2_**, the orbital contours at the HOMO and the HOMO–1 are consistent with those of **1-CO**–**3-CO**, showing considerable contributions of the donor moiety (HOMO, 60.8–68.5%; HOMO–1, 38.8–41.9%) and the π-spacer (HOMO, 28.0–31.2%; HOMO–1, 39.4–55.7%). The LUMO is localized from the π-spacer (32.3–68.8%) to the acceptor moiety (20.1–64.9%), and no contribution of the dibutylmethylene bridging group at the π-spacer is found. As seen in **1-CO**–**3-CO**, the contribution of the acceptor moiety to the LUMO is more remarkable when the stronger acceptor is employed. As for the LUMO+1, the orbital contour is found mainly on the π-spacer (12.1–45.2%) and the acceptor moiety (14.2–86.1%), accompanied by some contribution aminophenyl moiety of the donor. Thus, the orbital contours of the LUMO and the LUMO+1 in **1-CO**–**3-CO** indicates that the carbonyl bridging group at the π-spacer makes a significant contribution to the electronic transition.

**Figure 5:**
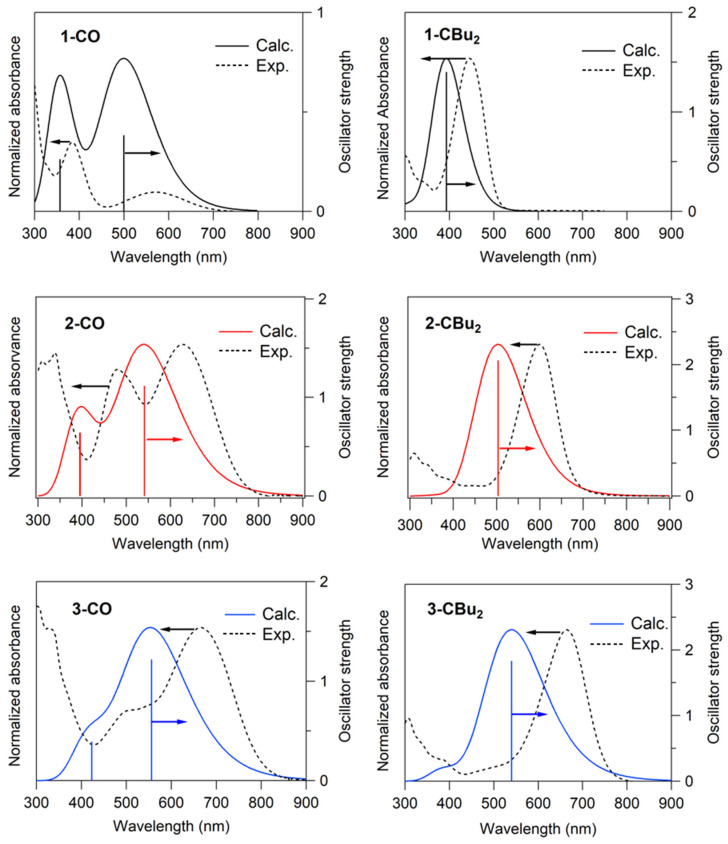
Calculated UV–vis–NIR absorption spectra (solid lines) of **1-CO**–**3-CO** and **1-CBu_2_**–**3-CBu_2_**. The corresponding experimental spectra are also shown together by dashed lines.

The vertical transitions were also calculated for **1-CO**–**3-CO** and **1-CBu_2_**–**3-CBu_2_**, and the results for the main transitions, from S_0_ to S_1_ and/or from S_0_ to S_2_, are summarized in Table 3. The spectroscopic simulations are also shown in Figure 5. In the case of **1-CO**–**3-CO**, the carbonyl bridging group in the π-spacer basically participates in the S_0_→S_1_ and S_0_→S_2_ transitions in the visible-to-near UV regions. The S_0_→S_1_ transition receives contributions mainly from the HOMO→LUMO and HOMO–1→LUMO transitions, showing the ICT character from the donor and π-spacer moieties to the acceptor moiety. The main components of the S_0_→S_2_ transition are the HOMO→LUMO+1 and HOMO–1→LUMO+1 transitions, and the ICT from the donor and π-spacer moieties to the carbonyl bridging group was involved, along with the ICT from the donor and the π-spacer to the acceptor. As shown in Figure 5, the overall spectral shapes of the simulations (the number of absorption bands in the visible-to-near UV regions and the oscillator strengths) are almost consistent with the experimental spectra, although the simulated spectra are somewhat blue-shifted. On the other hand, in the case of **1-CBu_2_**–**3-CBu_2_**, the main electronic transition exclusively consists of the S_0_→S_1_ transition contributed by HOMO→LUMO and HOMO–1→LUMO transitions; the ICT transition from the donor and π-spacer moieties to the acceptor moiety. The spectral simulations show good consistency with the experimental results, affording a single electronic transition in the visible region with a large oscillator strength, although the calculated transition energy is a little bit higher than the experimental one. Therefore, the panchromatic properties of the carbonyl-bridged dyes, especially **2-CO** and **3-CO**, are obviously attributed to the ICT transitions from the donor and π-spacer moieties to the acceptor moiety and the carbonyl bridging group.

### 2.5. Electrochemical Properties

**Figure 6:**
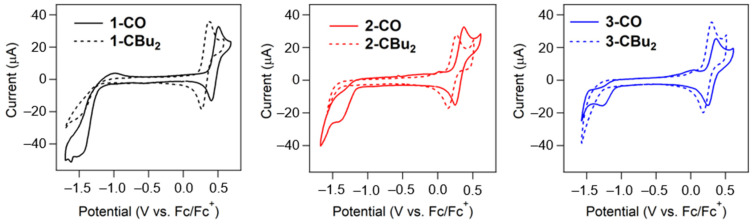
Cyclic voltammograms of **1-CO**–**3-CO** and **1-CBu_2_**–**3-CBu_2_** in dichloromethane. Tetrabutylammonium perchlorate (0.1 M) was used as a supporting electrolyte, and the potentials were recorded relative to an Ag/AgNO_3_ (0.1 M) reference electrode.

The electrochemical properties of the dyes were studied by cyclic voltammetry in dichloromethane. The cyclic voltammograms are shown in Figure 6 and the pertinent data are summarized in Table 4. All the compounds exhibited pseudo-reversible oxidation and reduction cycles on the oxidation side. The energy levels of HOMO (*E*_HOMO_) were calculated from their half-wave potentials (*E*_1/2, ox_) by comparing with that of the ferrocene/ferrocenium (Fc/Fc^+^) redox cycle (*E*_HOMO_; 4.80 eV). Any explicit reduction peaks were not observed in the reductive cycle for all the dyes. Thus, the LUMO level of each dye was estimated from the HOMO value and the optical band gap derived from the absorption spectral onset in the UV-vis-NIR spectrum. For **1-CO**–**3-CO**, the *E*_HOMO_ was determined approximately as −5.25 eV, independent of the structure of the acceptor moiety. On the other hand, the *E*_LUMO_ ranged from −3.53 eV for **1-CO** to −3.72 eV for **3-CO**, and the stronger acceptor moiety brought about the more stabilized LUMO. A similar trend of the *E*_HOMO_/*E*_LUMO_ values were found for **1-CBu_2_**–**3-CBu_2_**. That is, the HOMO was almost the same among the three dyes (−5.12 eV for **1-CBu_2_** and **3-CBu_2_** and −5.14 eV for **3-CBu_2_**), whereas the *E*_LUMO_ ranged from −2.66 eV for **1-CBu_2_** to −3.48 eV for **3-CBu_2_**. Obviously, the carbonyl-bridged structure is effective to stabilize the frontier orbitals: both of HOMO and LUMO of the carbonyl-bridged dyes (**1-CO**–**3-CO**) are more stabilized than the corresponding dibutylmethylene-bridged dyes (**1-CBu_2_**–**3-CBu_2_**). The impacts of the bridging group and the acceptor moiety on the *E*_HOMO_ and the *E*_LUMO_ are consistent with those obtained by the theoretical calculations, as shown in Supplementary Materials (Table S1): Both of the HOMO and LUMO levels of the carbonyl-bridged dyes are more stabilized in comparison with the corresponding dibutylmethylene-bridged dyes, and the stronger acceptor moiety leads to the stabilization of the LUMO level, whereas the HOMO level is almost constant among the dyes with the same bridging group.

### 2.6. Photostability Test

**Figure 7:**
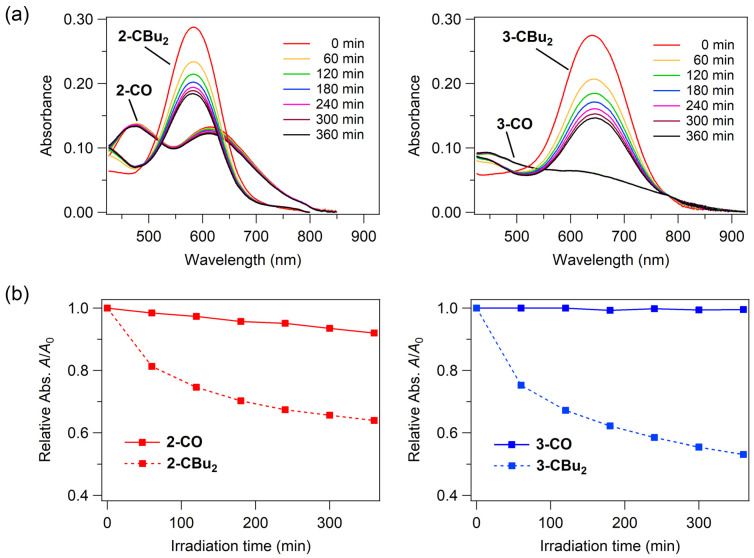
Time-course changes in (**a**) UV–vis–NIR absorption spectra and (**b**) relative absorbances (*A*/*A*_0_s) of **2-CO**-, **2-CBu_2_**-, **3-CO**-, and **3-CBu_2_**-doped PMMA films upon continuous irradiation of AM-1.5G light (Xe lamp: 100 mW cm^−2^). The doping level of the dye-doped PMMA film; methyl methacrylate monomer unit/dye = 99/1 (mol/mol). The values of *A*/*A*_0_ were monitored at 612, 584, 667, and 641 nm for **2-CO**, **2-CBu_2_**, **3-CO**, and **3-CBu_2_**, respectively.

The photostability of **2-CO**, **3-CO**, **2-CBu_2,_** and **3-CBu_2_** were evaluated in poly(methyl methacrylate) (PMMA) film under irradiation with a Xe lamp (AM-1.5G solar simulator; power: 100 mW cm^−2^). Figure 7a illustrates the time evolution of the UV-vis-NIR absorption spectra of the dyes in a PMMA matrix (methacrylate monomer unit/dye, 99/1, mol/mol). The PMMA films doped with the dyes were prepared by a spin-coating method, using a chloroform ink solution. The sample films were covered with glass caps in a glove box to keep them away from oxygen and moisture. It was found that the absorbances of **2-CO**- and **3-CO**-doped films hardly changed over the period of continuous light irradiation, indicating that the carbonyl-bridged dyes showed high photostability. In comparison, the absorbances of **2-CBu_2_**- and **3-CBu_2_**-doped films decreased dramatically after light irradiation. The relative absorbances (*A*/*A*_0_s) of **2-CO**, **2-CBu_2_** and **3-CO**, **3-CBu_2_** upon light irradiation are shown in Figure 7b, where the time-course changes were monitored at 612, 584, 667, and 641 nm for **2-CO**, **2-CBu_2_**, **3-CO**, and **3-CBu_2_**, respectively. A slight decrease in *A*/*A*_0_ was observed in 6 h for **2-CO**- and **3-CO**-doped films; *A*/*A*_0_ = 0.92 and 0.997, respectively. On the other hand, considerable bleaching was observed in the same period for **2-CBu_2_**- and **3-CBu_2_**-doped films; *A*/*A*_0_ = 0.64 and 0.53, respectively. From these results, **2-CO** and **3-CO** have higher stability against photoirradiation in comparison with the corresponding reference dyes **2-CBu_2_** and **3-CBu_2_**. The high photostability should be attributed to stabilization of the frontier orbitals by introduction of the carbonyl bridging group in the bithiophene π-spacer.

## 3. Methods

### 3.1. Spectroscopic Measurements

UV–vis–NIR absorption spectra were measured on a Shimadzu (Kyoto, Japan) UV3600 spectrophotometer. PL spectra were measured on a Horiba (Kyoto, Japan) Jobin Yvon SPEX Fluorolog-3 spectrofluorometer. PL quantum yields were measured on a Hamamatsu Photonics (Hamamatsu, Japan) C13534-01 absolute PL quantum yield measurement system. PL lifetimes were obtained on a Horiba (Kyoto, Japan) Jobin Yvon FluoroCube spectroanalyzer. The sample solutions for spectral data acquisition were prepared by using solvents of spectroscopic grade. The concentration of the samples was adjusted to 1 × 10^−5^ M for UV–vis–NIR absorption spectroscopy. For PL spectra, the concentration was adjusted to 1 × 10^−6^ M.

### 3.2. Measurements of Electrochemical Properties

Cyclic voltammograms of the dyes were recorded on a Hokuto Denko (Tokyo, Japan) HZ-5000 electrochemical measurement system at a scanning rate of 100 mV s^−1^. The measurements were conducted in deaerated dichloromethane (dye concentration; 1 mM), where 0.1 M tetrabutylammonium perchlorate was used as a supporting electrolyte. The potential was recorded relative to an Ag/AgNO_3_ (0.1 M) reference electrode with a Pt wire being used for both working and counter electrodes.

### 3.3. Photostability Tests

The photostability of the dyes was evaluated by considering the changes in the absorption spectra over the photoirradiation time upon exposure to a Xe lamp, where an AM-1.5G solar simulator (power: 100 mW cm^−2^; CEP-2000, Bunko-Keiki, Hachioji, Japan) was used as a light source. The dye-doped poly(methyl methacrylate) (PMMA) film samples were irradiated at ambient temperature under identical irradiation conditions. For preparation of the film sample, a chloroform solution containing PMMA and the dye (methyl methacrylate monomer unit/dye, 99/1, mol/mol) was spin-coated (slope, 5.0 s, then 1000 rpm, 60 s) on a glass plate, and the obtained film was dried at 60 °C for 15 min. The film sample for each dye was covered with a glass cap and encapsulated by using a UV-curing epoxy resin in a glove box filled with dry argon. The absorption spectra of the film samples were measured for 360 min at 60 min interval during the photoirradiation.

## 4. Conclusions

In summary, novel D–π–A-type molecules bearing bridged bithiophene π–spacers, **1-CO**–**3-CO** and **1-CBu_2_**–**3-CBu_2_**, were successfully synthesized, and their photophysical properties were evaluated. The dyes **2-CO** and **3-CO** showed panchromatic absorption spectra consisting of two absorption peaks in the visible region. DFT/TD-DFT calculations suggested that the absorption spectra of **2-CO** and **3-CO** involved two electronic transitions in the visible region: one is the transition mainly from the donor to the acceptor, and the other from the donor to the carbonyl bridging group in the bithiophene π-spacer. Furthermore, **2-CO** and **3-CO** exhibited weak NIR emissions at 925 and 937 nm, respectively. The less emissive behavior should be attributed to the intersystem crossing from the singlet excited state to the triplet state facilitated by the carbonyl-bridged structure, according to the El-Sayed’s rule. On the other hand, the referential dyes with the dibutylmethylene bridging group, **2-CBu_2_** and **3-CBu_2_**, had single electronic transition bands in the visible region with large molar absorption coefficients and exhibited relatively intense NIR fluorescence emission. The results of the photostability tests for **2-CO** and **3-CO** in a PMMA matrix showed that the carbonyl-bridged structure led to the stabilization of frontier orbitals, resulting in higher photostability compared to the corresponding dibutylmethylene-bridged reference dyes.

## Figures and Tables

**Table 1:** Optical properties of **1-CO**–**3-CO** and **1-CBu_2_**–**3-CBu_2_** in dichloromethane at room temperature.

Compd	λ_abs_ [nm] (ε_abs_ [mol^−1^ L cm^−1^])	Compd	λ_abs_ [nm] (ε_abs_ [mol^−1^ L cm^−1^])
**1-CO**	285 (61,800), 383 (27,900), 569 (7800)	**1-CBu_2_**	296 (16,900), 445 (45,800)
**2-CO**	338 (29,800), 481 (24,300), 631 (31,500)	**2** **-CBu_2_**	308 (24,500), 598 (87,200)
**3-CO**	328 (34,500), 498 (15,700), 667 (34,600)	**3** **-CBu_2_**	307 (32,900), 665 (77,500)

**Table 2:** Photoluminescence and photophysical properties of **1-CO**–**3-CO** and **1-CBu_2_**–**3-CBu_2_** in dichloromethane at room temperature.

Compd	λ_PL_ (nm)	*Φ*_PL_	$\Delta \tilde{\nu }$ (cm^−1^)	τ_PL_ (ns)	*k*_r_ (10^7^ s^−1^)	*k*_nr_ (10^7^ s^−1^)
**1** **-CO**	--- ^1^	--- ^1^	--- ^1^	--- ^1^	--- ^1^	--- ^1^
**2-CO**	926	<0.01	5120	--- ^2^	--- ^2^	--- ^2^
**3-CO**	937	<0.01	4320	--- ^2^	--- ^2^	--- ^2^
**1-CBu_2_**	592	0.93	5440	2.84	32.7 ^3^	2.46 ^3^
**2** **-CBu_2_**	742	0.48	3190	1.95	24.6 ^3^	26.7 ^3^
**3** **-CBu_2_**	823	0.03	2860	--- ^2^	--- ^2^	--- ^2^

^1^ Not determined due to no emission. ^2^ Not determined due to very weak emission. ^3^ *k*_r_ = *Φ*_PL_/τ_PL_. *k*_nr_ = (1 – *Φ*_PL_)/τ_PL_.

**Table 3:** Calculated molecular orbital components, absorption maxima (λ_abs, calc_s), and oscillator strengths (*f*s) for main electronic transitions of **1-CO**–**3-CO** and **1-CBu_2_**–**3-CBu_2_**.

Compd	Transition	Component	λ_abs,calc_ (*f*)
**1-CO**	S_0_ → S_1_	HOMO → LUMO (60%) HOMO−1 → LUMO (35%)	499.70 nm (0.3829)
	S_0_ → S_3_ ^1^	HOMO → LUMO (41%) HOMO−1 → LUMO (31%)	356.84 nm (0.3404)
**1** **-CBu_2_**	S_0_ → S_1_	HOMO → LUMO (79%) HOMO−1 → LUMO (15%)	393.24 nm (1.401)
**2-CO**	S_0_ → S_1_	HOMO → LUMO (62%) HOMO−1 → LUMO (25%)	540.97 nm (1.116)
	S_0_ → S_2_	HOMO−1 → LUMO+1 (38%) HOMO → LUMO+1 (32%)	395.36 nm (0.6429)
**2** **-CBu_2_**	S_0_ → S_1_	HOMO → LUMO (73%) HOMO−1 → LUMO (22%)	503.28 nm (2.063)
**3-CO**	S_0_ → S_1_	HOMO → LUMO (56%) HOMO−1 → LUMO (25%)	556.05 nm (1.217)
	S_0_ → S_2_	HOMO → LUMO+1 (33%) HOMO−1 → LUMO+1 (28%)	423.17 nm (0.3909)
**3** **-CBu_2_**	S_0_ → S_1_	HOMO → LUMO (69%) HOMO−1 → LUMO (24%)	540.19 nm (1.833)

^1^ The oscillator strength of the S_0_−S_2_ transition of **1-CO** is approximately zero, and thus the second transition band is assigned to the S_0_−S_3_ transition.

**Table 4:** Electrochemical data (half-wave oxidation potentials *E*_1/2, ox_s), HOMO and LUMO energy levels (*E*_HOMO_s and *E*_LUMO_s, respectively), and optical band gaps (*E*_opt_s) of **1-CO**–**3-CO** and **1-CBu_2_**–**3-CBu_2_**.

Compd	*E*_1/2, ox_ (V) vs. Fc/Fc^+^	*E*_HOMO_ ^1^ (eV)	*E*_LUMO_ ^2^ (eV)	*E*_opt_ ^3^ (eV)
**1-CO**	0.450	−5.25	−3.53	1.72
**1-CBu_2_**	0.315	−5.12	−2.66	2.46
**2-CO**	0.455	−5.26	−3.63	1.63
**2-CBu_2_**	0.335	−5.14	−3.32	1.82
**3-CO**	0.445	−5.25	−3.72	1.53
**3-CBu_2_**	0.320	−5.12	−3.48	1.64

^1^*E*_HOMO_ (eV) = −(*E*_1/2, ox_ + 4.80). ^2^ *E*_LUMO_ (eV) = *E*_HOMO_ + *E*_opt_. ^3^ *E*_opt_ (eV) = 1240/λ_onset_, where λ_onset_ represents the onset of a UV-vis-NIR spectrum.

## Data Availability

The original data presented in this study are included in the main text and the Supplementary Materials. Further inquiries can be directed to the corresponding author.

## Supplementary Materials

The following supporting information can be downloaded at: https://www.mdpi.com/article/10.3390/molecules30153084/s1, Materials and Methods; Figure S1: ^1^H and ^13^C NMR spectra of **1-CO**; Figure S2: ^1^H and ^13^C NMR spectra of **2-CO**; Figure S3: ^1^H NMR spectrum of **3-CO**; Figure S4: ^1^H and ^13^C NMR spectra of **1-CBu_2_**; Figure S5: ^1^H and ^13^C NMR spectra of **2-CBu_2_**; Figure S6: ^1^H and ^13^C NMR spectra of **3-CBu_2_**; Figure S7: UV-vis-NIR spectra of **1-CO**, **1-CBu_2_**, **2-CO**, **2-CBu_2_**, **3-CO**, and **3-CBu_2_** in various solvents (acetone, dichloromethane, chloroform, and toluene) at room temperature; Figure S8: Photoluminescence spectra of **1-CBu_2_**, **2-CO**, **2-CBu_2_**, **3-CO**, and **3-CBu_2_** in various solvents (acetone, dichloromethane, chloroform, and toluene) at room temperature; Table S1: Calculated HOMO and LUMO energy levels and HOMO/LUMO gaps of **1-CO**–**3-CO** and **1-CBu_2_**–**3-CBu_2_**; Table S2: Contribution of donor, π-spacer, and acceptor units to molecular orbitals in **1-CO**–**3-CO** and **1-CBu_2_**–**3-CBu_2_**.
