# Supplementary material for: Synthesis and Electronic Properties of Novel Donor–π–Acceptor-Type Functional Dyes with a Carbonyl-Bridged Bithiophene π-Spacer
Source: Molecules. 2025 Jul 23;30(15):3084. doi: 10.3390/molecules30153084 (PMC12348573; doi:10.3390/molecules30153084)
Supplement: Supplementary file 1 [file molecules-30-03084-s001.zip › molecules-3725933-supplementary.pdf]

Supplementary Materials

Synthesis and Electronic Properties of Novel Donor–π–Acceptor-Type Functional Dyes with a Carbonyl-Bridged Bithiophene π-Spacer

Miyu Ueda, Ryo Nagayama, Masaki Nagaoka, Naoya Suzuki, Shintaro Kodama, Takeshi Maeda, Shin-ichiro Kato and Shigeyuki Yagi *

Department of Applied Chemistry, Graduate School of Engineering, Osaka Metropolitan University, 1-1 Gakuen-cho, Naka-ku, Sakai, Osaka 599-8531, Japan

* Correspondence: yagi@omu.ac.jp

Table of Contents

1. Materials and Methods: Synthesis of the Dyes

1.1. Synthesis of 6-(4-(diphenylamino)phenyl)-4-oxo-4*H*-cyclopenta[2,1-*b*:3,4-*b*']dithiophene-2-carbaldehyde (**1-CO**)

1.2. Synthesis of 5-((6-(4-(diphenylamino)phenyl)-4-oxo-4*H*-cyclopenta[2,1-*b*:3,4-*b*']dithiophen-2-yl)methylene)-1,3-diethyl-2-thioxodihydropyrimidine-4,6(1*H*,5*H*)-dione (**2-CO**)

1.3. Synthesis of 2-(2-((6-(4-(diphenylamino)phenyl)-4-oxo-4*H*-cyclopenta[2,1-*b*:3,4-*b*']dithiophen-2-yl)methylene)-3-oxo-2,3-dihydro-1*H*-inden-1-ylidene)malononitrile (**3-CO**)

1.4. Synthesis of 4,4-dibutyl-6-(4-(diphenylamino)phenyl)-4*H*-cyclopenta[2,1-*b*:3,4-*b*']dithiophene-2-carbaldehyde (**1-CBu$_2$**)

1.5. Synthesis of 5-((4,4-dibutyl-6-(4-(diphenylamino)phenyl)-4*H*-cyclopenta[2,1-*b*:3,4-*b*']dithiophen-2-yl)methylene)-1,3-diethyl-2-thioxodihydropyrimidine-4,6(1*H*,5*H*)-dione (**2-CBu$_2$**)

1.6. Synthesis of 2-(2-((4,4-dibutyl-6-(4-(diphenylamino)phenyl)-4*H*-cyclopenta[2,1-*b*:3,4-*b*']dithiophen-2-yl)methylene)-3-oxo-2,3-dihydro-1*H*-inden-1-ylidene)malononitrile (**3-CBu$_2$**)

2. ^{1}H and ^{13}C NMR Spectra

Figure S1: ^{1}H and ^{13}C NMR spectra of **1-CO**

Figure S2: ^{1}H and ^{13}C NMR spectra of **2-CO**

Figure S3: ^{1}H NMR spectrum of **3-CO**

Figure S4: ^{1}H and ^{13}C NMR spectra of **1-CBu$_2$**

Figure S5: ^{1}H and ^{13}C NMR spectra of **2-CBu$_2$**

Figure S6: ^{1}H and ^{13}C NMR spectra of **3-CBu$_2$**

3. Solvatochromic Properties

Figure S7: UV-vis-NIR spectra of **2-CO**, **3-CO**, and **1-CBu$_2$–3-CBu$_2$** in various solvents at room temperature.

Figure S8: Photoluminescence spectra of **1-CO–3-CO** and **1-CBu$_2$–3-CBu$_2$** in various solvents at room temperature.

4. Theoretical Calculations

Table S1: Calculated HOMO and LUMO energy levels ($E_{HOMO, calc}$ and $E_{LUMO, calc}$, respectively) and HOMO/LUMO gaps ($\Delta E_{H–L, calc}$) of **1-CO–3-CO** and **1-CBu$_2$–3-CBu$_2$** (M062X/6-31G(d,p) level of theory with the SMD solvation model in dichloromethane).

Table S2: Contribution of donor, π-spacer, and acceptor units to molecular orbitals in **1-CO–3-CO** and **1-CBu$_2$–3-CBu$_2$** obtained by the method in the literature (Lu, T.; Chen, F. Multiwfn: A Multifunctional Wavefunction Analyzer. *J. Comput. Chem.*, **2012**, *33*, 580–592).

1. Synthesis of the dyes

The synthetic procedures of the dyes are described in detail in the Supplementary Materials. The reagents, the catalysts, and the solvents were purchased from FUJIFILM Wako Pure Chemical Corporation (Osaka, Japan), Tokyo Chemical Industry Co., Ltd. (Tokyo, Japan), Kanto Chemical Co., Inc. (Tokyo, Japan), AmBeed Co., Inc. (Illinois, USA), or BLD Pharmatech Ltd. (Shanghai, China) and used without further purification. ^{1}H NMR (400 MHz) and ^{13}C NMR (101 MHz) spectra were obtained on a JEOL (Akishima, Japan) ECX-400 spectrometer, using TMS (0.00 ppm for ^{1}H and ^{13}C NMR) as an internal standard. Electrospray ionization time-of-flight mass spectra (ESI-TOF-MS) were measured on a JEOL (Akishima, Japan) JMS-T100LP mass spectrometer. Elemental analyses were carried out on a J-Science Lab (Kyoto, Japan) MICRO CORDER JM10 analyzer.

1.1. Synthesis of 6-(4-(diphenylamino)phenyl)-4-oxo-4*H*-cyclopenta[2,1-*b*:3,4-*b*']dithiophene-2-carbaldehyde (**1-CO**)

Tripotassium phosphate (567 mg, 2.67 mmol), 6-bromo-4-oxo-4*H*-cyclopenta[2,1-*b*:3,4-*b*']dithiophene-2-carbaldehyde (**4a**, 400 mg, 1.34 mmol), 4-(diphenylamino)phenylboronic acid (387 mg, 1.34 mmol), and tetrakis(triphenylphosphine)palladium(0) (77 mg, 0.067 mmol) were added to a mixture of 1,2-dimethoxyethane/ethanol/water (7 mL/1 mL/2 mL) under nitrogen atmosphere. The mixture was heated to 80 °C for 18 h. After cooling down to room temperature, water (50 mL) was added to the mixture, and the crude product was extracted with dichloromethane (3 × 50 mL). The organic layers were combined and washed with brine (50 mL). The organic solution was separated and dried over anhydrous magnesium sulfate ($MgSO_4$). After the solvent was removed under reduced pressure on a rotary evaporator, the crude product was purified by silica gel column chromatography (dichloromethane/ethyl acetate = 50/1, v/v) to yield **1-CO** as a dark purple solid (550 mg, 89%); ^{1}H NMR (400 MHz, $CDCl_3$) δ 9.77 (s, 1H), 7.49 (d, *J* = 8.7 Hz, 2H), 7.59 (s, 1H), 7.29 (t, *J* = 7.8 Hz, 4H), 7.19 (s, 1H), 7.15–7.09 (m, 6H), 7.06 (d, *J* = 8.7 Hz, 2H); ^{13}C NMR (101 MHz, $CDCl_3$) δ 182.09, 181.80, 158.71, 152.16, 148.80, 147.07, 145.93, 145.26, 144.23, 141.16, 129.59, 126.47, 126.32, 125.19, 123.93, 122.67, 116.34; ESI-TOF-MS (*m/z*) calcd for $C_{28}H_{17}NO_2S_2$ (M^+): 463.09. Found: 463.09. Anal. Calcd for $C_{28}H_{17}NO_2S_2$: C, 72.55; H, 3.70; N, 3.02. Found: C, 72.68; H, 3.52; N, 2.90.

1.2. Synthesis of 5-((6-(4-(diphenylamino)phenyl)-4-oxo-4*H*-cyclopenta[2,1-*b*:3,4-*b*']dithiophen-2-yl)methylene)-1,3-diethyl-2-thioxodihydropyrimidine-4,6(1*H*,5*H*)-dione (**2-CO**)

Triethylamine (99 μL, 0.72 mmol), **1-CO** (139 mg, 0.216 mmol), and 1,3-diethyl-2-thiobarbituric acid (90.0 mg, 0.449 mmol) were dissolved in dichloromethane (3 mL), and the mixture was stirred at room temperature for 17 h under nitrogen atmosphere. Then, water (50 mL) was added and the resultant mixture was extracted with chloroform (3 × 50 mL). The combined organic layers were washed with brine (50 mL) and dried over anhydrous $MgSO_4$. The solvent was removed under reduced pressure on a rotary evaporator, and the crude product was purified by silica gel column chromatography (dichloromethane/ethyl acetate = 50/1, v/v) to yield **2-CO** as a black solid (112 mg,

81%); ^{1}H NMR (400 MHz, $CDCl_3$) δ8.55 (s, 1H), 7.65 (s, 1H), 7.42 (d, *J* = 8.7 Hz, 2H), 7.29 (t, *J* = 7.8 Hz, 4H), 7.22 (s, 1H), 7.14–7.03 (m, 6H), 7.05 (d, *J* = 8.7 Hz, 2H), 4.61–4.54 (m, 4H), 1.35–1.28 (m, 6H); ^{13}C NMR (101 MHz, $CDCl_3$) δ181.10, 178.45, 165.41, 160.76, 160.44, 154.57, 149.18, 148.91, 147.64, 146.91, 145.39, 141.94, 140.09, 137.79, 129.63, 126.64, 125.95, 125.35, 124.14, 122.32, 116.37, 109.14, 44.08, 43.32, 12.57, 12.43; ESI-TOF-MS (*m/z*) calcd for $C_{36}H_{27}N_3O_3S_3+K^+$ ($[M + K]^+$): 684.09. Found: 684.84. Anal. Calcd for $C_{36}H_{27}N_3O_3S_3$: C, 66.95; H, 4.21; N, 6.51. Found: C, 66.95; H, 4.23; N, 6.35.

1.3. Synthesis of 2-(2-((6-(4-(diphenylamino)phenyl)-4-oxo-4*H*-cyclopenta[2,1-*b*:3,4-*b*']dithiophen-2-yl)methylene)-3-oxo-2,3-dihydro-1*H*-inden-1-ylidene)malononitrile (**3-CO**)

1,4-Diazablicyclo[2.2.2]octane (110 mg, 0.984 mmol), **1-CO** (80.0 mg, 0.173 mmol), and 3-(dicyanomethylidene)indan-1-one (87.0 mg, 0.449 mmol) were dissolved in dichloromethane (3 mL), and the mixture was stirred at room temperature for 22 h under nitrogen atmosphere. Then, water (50 mL) was added and the resultant mixture was extracted with chloroform (3 × 50 mL). The combined organic layers were washed with brine (50 mL) and dried over anhydrous $MgSO_4$. The solvent was removed under reduced pressure on a rotary evaporator, and the residue was washed with ethanol and hexane. The purified solid was collected by filtration to afford **3-CO** as a black solid (53.9 mg, 49%); ^{1}H NMR (400 MHz, $CDCl_3$) δ8.76 (s, 1H), 8.66 (d, *J* = 7.7 Hz, 1H), 7.89 (d, *J* = 7.3 Hz, 1H), 7.78–7.71 (m, 2H), 7.63 (s, 1H), 7.41 (d, *J* = 8.7 Hz, 2H), 7.30 (t, *J* = 7.3 Hz, 4H), 7.19 (s, 1H), 7.15-7.08 (m, 6H), 7.94 (d, *J*=8.7 Hz, 2H); ESI-TOF-MS (*m/z*) calcd for $C_{40}H_{21}N_3O_2S_2+Na^+$ ($[M + Na]^+$): 662.09. Found: 662.87. Anal. Calcd for $C_{40}H_{21}N_3O_2S_2$: C, 75.10; H, 3.31; N, 6.57. Found: C, 74.74; H, 3.55; N, 6.33. The ^{13}C NMR spectrum of **3-CO** was not obtained due to low solubility in any deuterated solvents usually used for NMR measurements.

1.4. Synthesis of 4,4-dibutyl-6-(4-(diphenylamino)phenyl)-4*H*-cyclopenta[2,1-*b*:3,4-*b*']dithiophene-2-carbaldehyde (**1-CBu$_2$**)

The dye **1-CBu$_2$** was prepared according to the same method for the preparation of **1-CO**, where 6-bromo-4,4-dibutyl-4*H*-cyclopenta[2,1-*b*:3,4-*b*']dithiophene-2-carbaldehyde (**4b**) was used as a starting material in place of **3**; Yield, 88%; ^{1}H NMR (400 MHz, $CDCl_3$) δ 9.81 (s, 1H), 7.54 (s,1H), 7.49 (d, *J* = 9.1 Hz, 2H), 7.27 (t, *J* = 7.3 Hz, 4H), 7.13–7.10 (m, 5H), 7.09–7.03 (m, 4H), 1.94–1.82 (m, 4H), 1.22–1.12 (m, 4H), 0.97–0.90 (m, 4H), 0.77 (t, *J* = 7.3 Hz, 6H); ^{13}C NMR (101 MHz, $CDCl_3$) δ 182.53, 163.61, 157.42, 149.57, 148.33, 147.92, 147.36, 142.92, 133.92, 130.02, 129.47, 128.29, 126.46, 124.77, 123.51, 123.47, 116.76, 54.09, 37.66, 26.81, 23.11, 14.01; ESI-TOF-MS (*m/z*) calcd for $C_{36}H_{35}NOS_2$ (M^+): 561.21. Found: 561.76; Anal. Calcd for $C_{36}H_{35}NOS_2$: C, 76.97; H, 6.28; N, 2.49. Found: C, 76.97; H, 6.48; N, 2.52.

1.5. Synthesis of 5-((4,4-dibutyl-6-(4-(diphenylamino)phenyl)-4*H*-cyclopenta[2,1-*b*:3,4-*b*']dithiophen-2-yl)methylene)-1,3-diethyl-2-thioxodihydropyrimidine-4,6(1*H*,5*H*)-dione (**2-**

CBu2)

The dye **2-CBu2** was prepared as a black solid according to a similar method for the preparation of **2-CO**, where **1-CBu2** was used as a starting material in place of **1-CO**; Yield, 91%: ^{1}H NMR (400 MHz, $CDCl_3$) δ 8.66 (s, 1H), 7.51 (d, *J* = 8.7 Hz, 2H), 7.28 (t, *J* = 7.3 Hz, 4H), 7.14–7.05 (m, 9H), 4.65–4.57 (m, 4H), 1.95–1.87 (m, 4H), 1.37–1.29 (m, 6H), 1.20–1.14 (m, 4H), 0.97–0.91 (m, 4H), 0.76 (t, *J* = 7.3 Hz, 6H); ^{13}C NMR (101 MHz, $CDCl_3$) δ 178.60, 167.11, 160.42, 153.96, 148.91, 148.64, 147.13, 138.95, 134.68, 129.55, 127.54, 126.77, 125.05, 123.81, 122.95, 116.78, 54.00, 43.92, 43.21, 37.75, 26.88, 23.07, 13.99, 12.64, 12.51; ESI-TOF-MS (*m/z*) calcd for $C_{44}H_{45}N_3O_2S_3$ ([M]$^+$): 743.32. Found: 743.28; Anal. Calcd for $C_{44}H_{45}N_3O_2S_3$: C, 71.03; H, 6.10; N, 5.65. Found: C, 71.05; H, 6.24; N, 5.40.

1.6. Synthesis of 2-(2-((4,4-dibutyl-6-(4-(diphenylamino)phenyl)-4*H*-cyclopenta[2,1-*b*:3,4-*b*']dithiophen-2-yl)methylene)-3-oxo-2,3-dihydro-1*H*-inden-1-ylidene)malononitrile (**3-CBu2**)

The dye **3-CBu2** was prepared as a black solid according to a similar method for the preparation of **3-CO**, where **1-CBu2** was used as a starting material in place of **1-CO**; Yield, 77%; ^{1}H NMR (400 MHz, $CDCl_3$) δ8.91 (s, 1H), 8.64 (d, *J* = 8.2 Hz, 1H), 7.86 (d, *J* = 5.9 Hz, 1H), 7.73-7.66 (m, 2H), 7.52 (d, *J* = 8.7 Hz, 2H), 7.29 (t, *J* = 7.7 Hz, 4H), 7.15–7.06 (m, 10H), 1.95–1.89 (m, 4H), 1.21–1.14 (m, 4H), 0.96–0.87 (m, 4H), 0.77 (t, *J* = 7.7 Hz, 6H); ^{13}C NMR (101 MHz, $CDCl_3$) δ 188.70, 167.25, 154.05, 148.67, 147.11, 140.06, 138.94, 138.22, 136.89, 134.77, 134.65, 133.99, 129.57, 127.51, 126.78, 125.09, 123.85, 123.36, 122.91, 116.83, 115.62, 115.48, 66.48, 54.10, 37.89, 26.84, 23.08, 14.00; ESI-TOF-MS (*m/z*) calcd for $C_{48}H_{39}N_3OS_3$ ([M]$^+$): 737.25. Found: 737.29. Anal. Calcd for $C_{48}H_{39}N_3OS_3$: C, 78.12; H, 5.33; N, 5.69. Found: C, 77.95; H, 5.19; N, 5.46.

2. ^{1}H and ^{13}C NMR Spectra

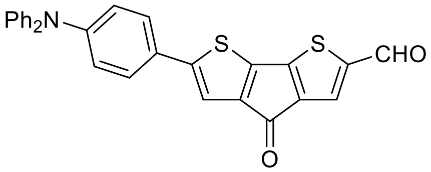

1-CO

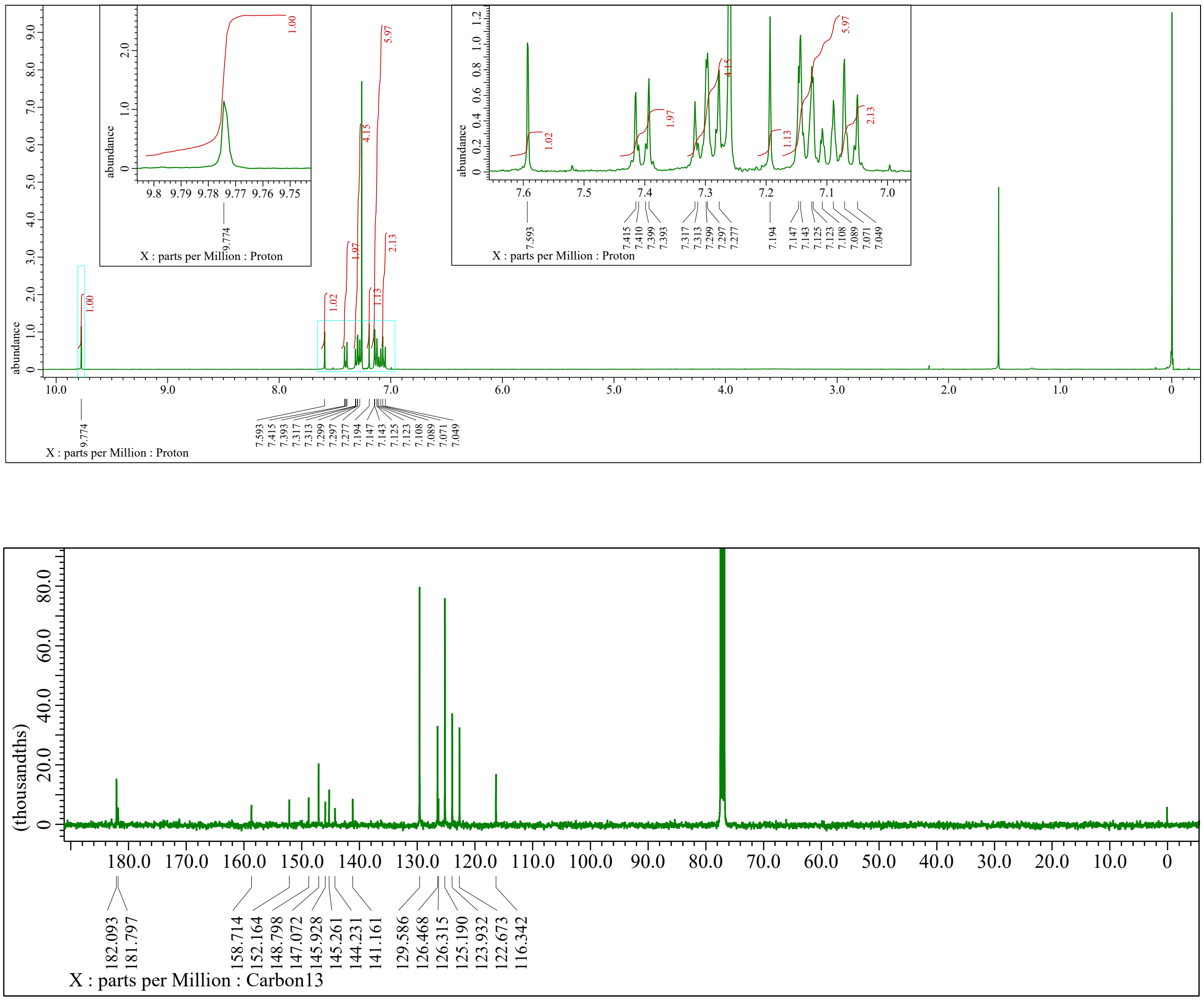

Figure S1. ^{1}H (upper, 400 MHz, $CDCl_3$) and ^{13}C (lower, 101 MHz, $CDCl_3$) NMR spectra of **1-CO**.

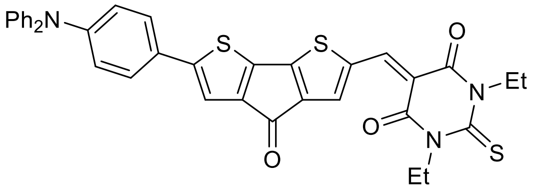

2-CO

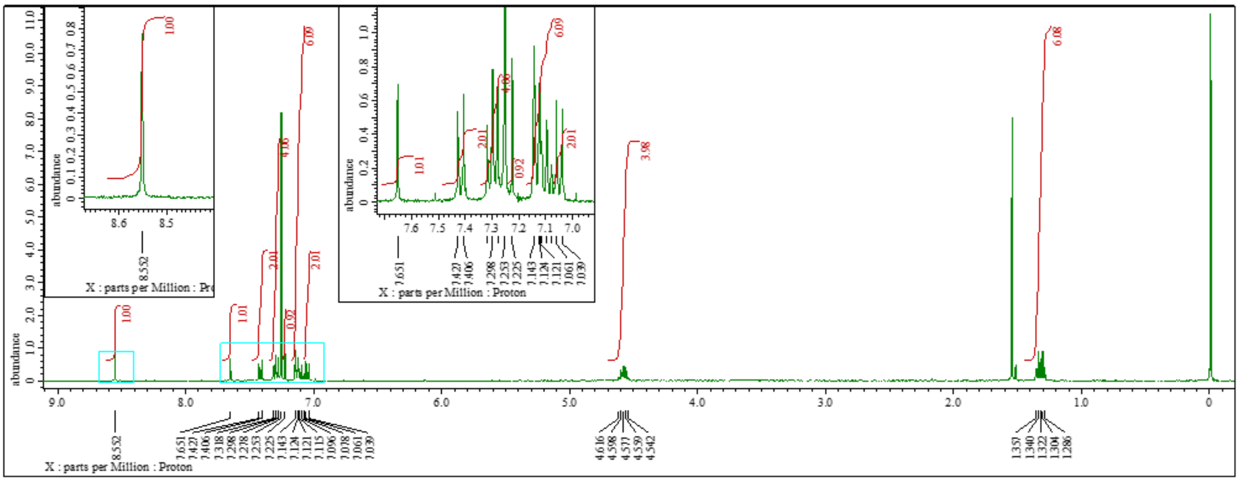

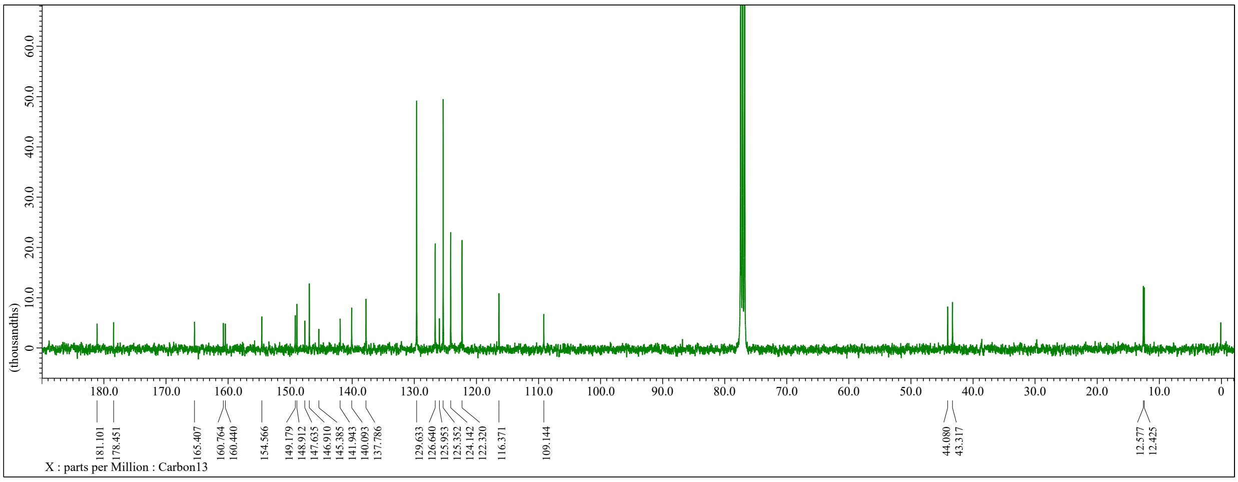

Figure S2. ^{1}H (upper, 400 MHz, $CDCl_3$) and ^{13}C (lower, 101 MHz, $CDCl_3$) NMR spectra of **2-CO**.

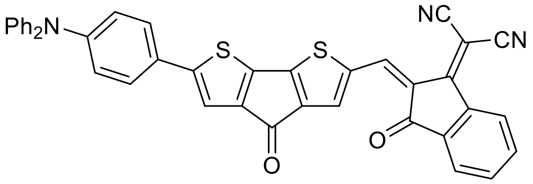

3-CO

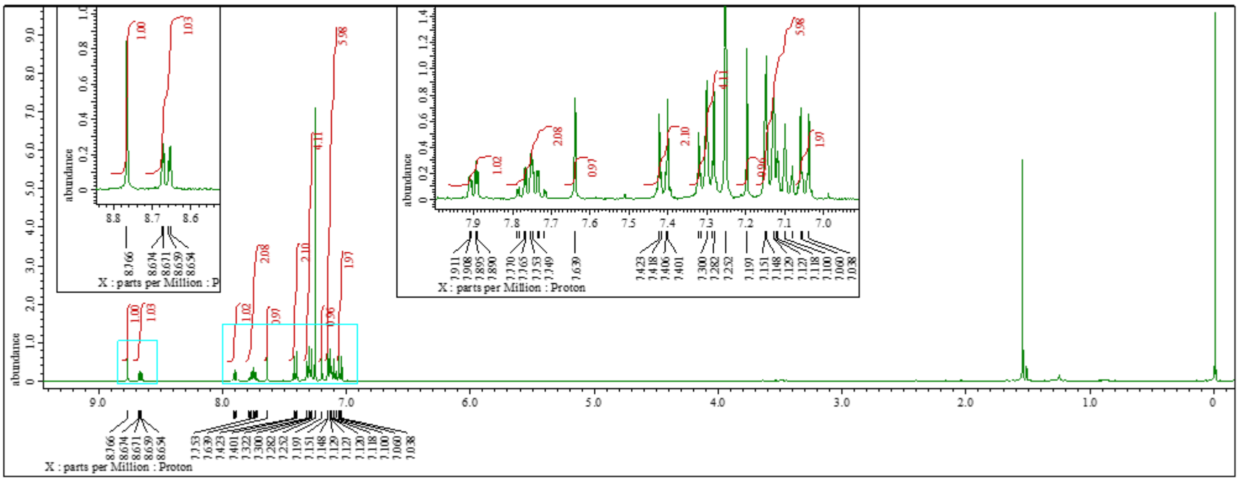

Figure S3. ^{1}H NMR spectrum of **3-CO** (400 MHz, $CDCl_3$).

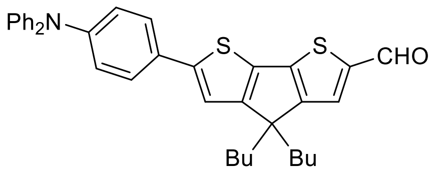

1-CBu$_2$

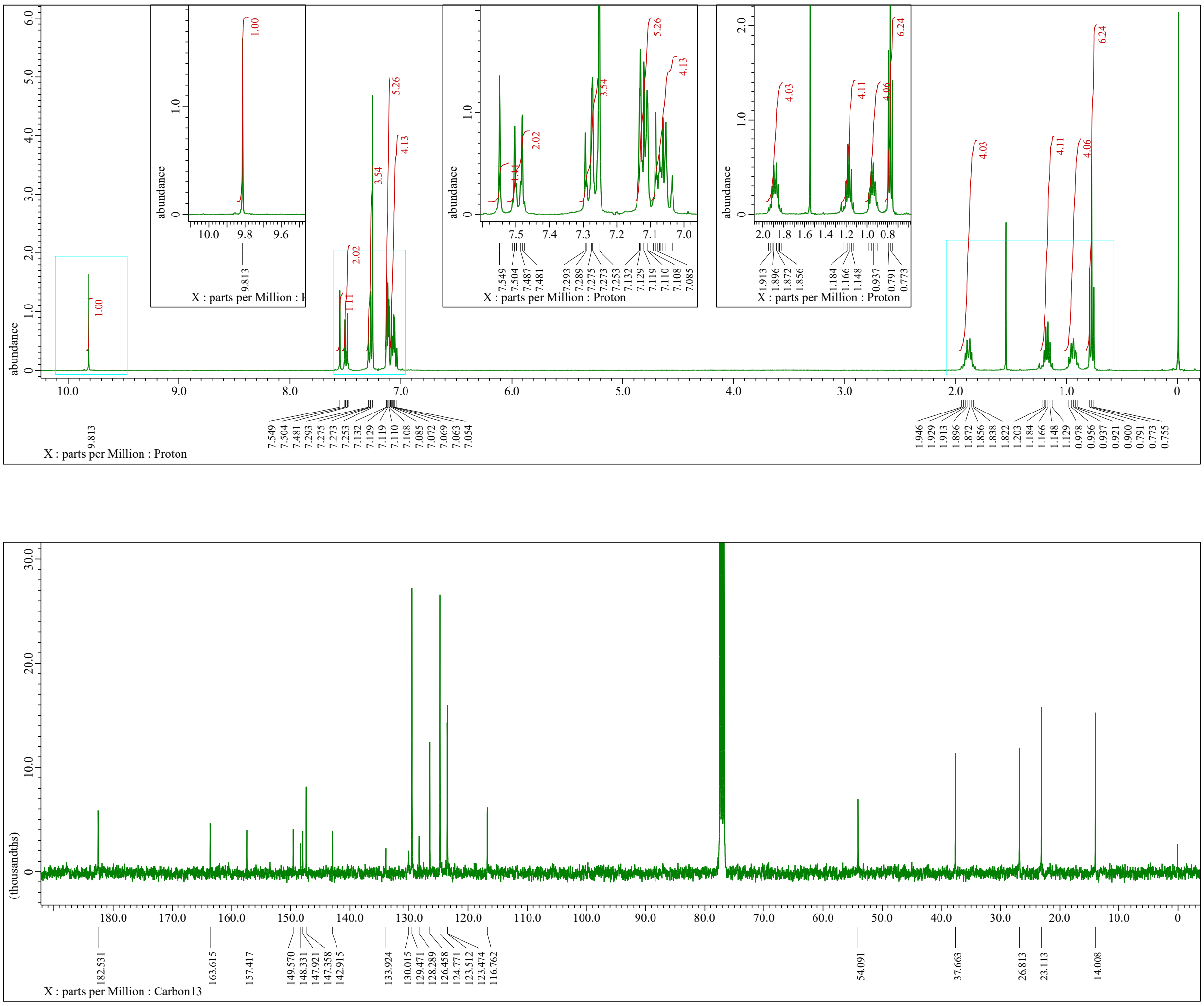

Figure S4. ^{1}H (upper, 400 MHz, $CDCl_3$) and ^{13}C (lower, 101 MHz, $CDCl_3$) NMR spectra of **1-CBu$_2$**.

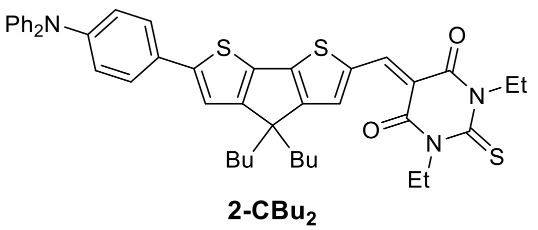

2-CBu$_2$

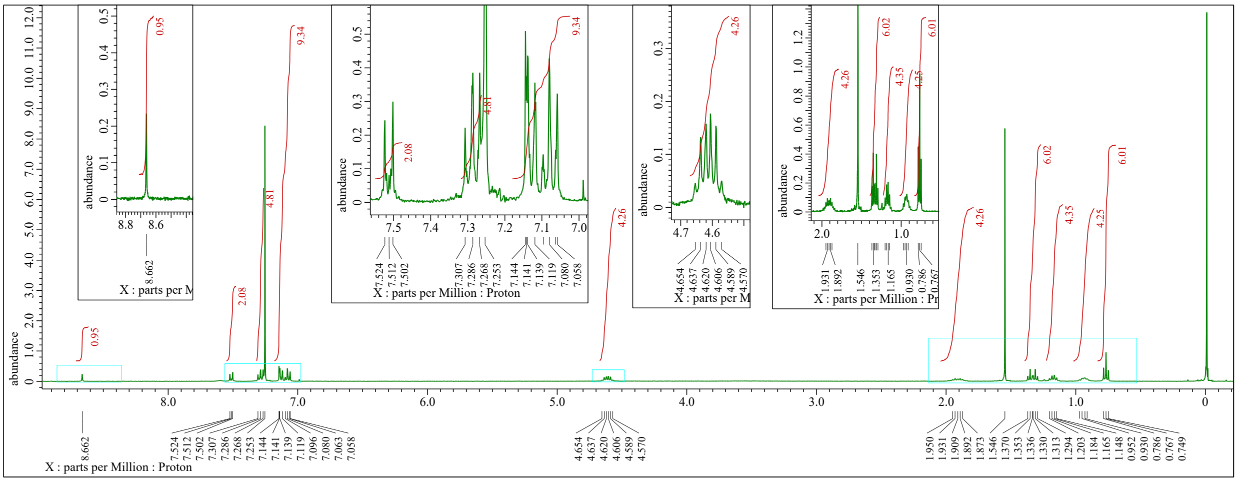

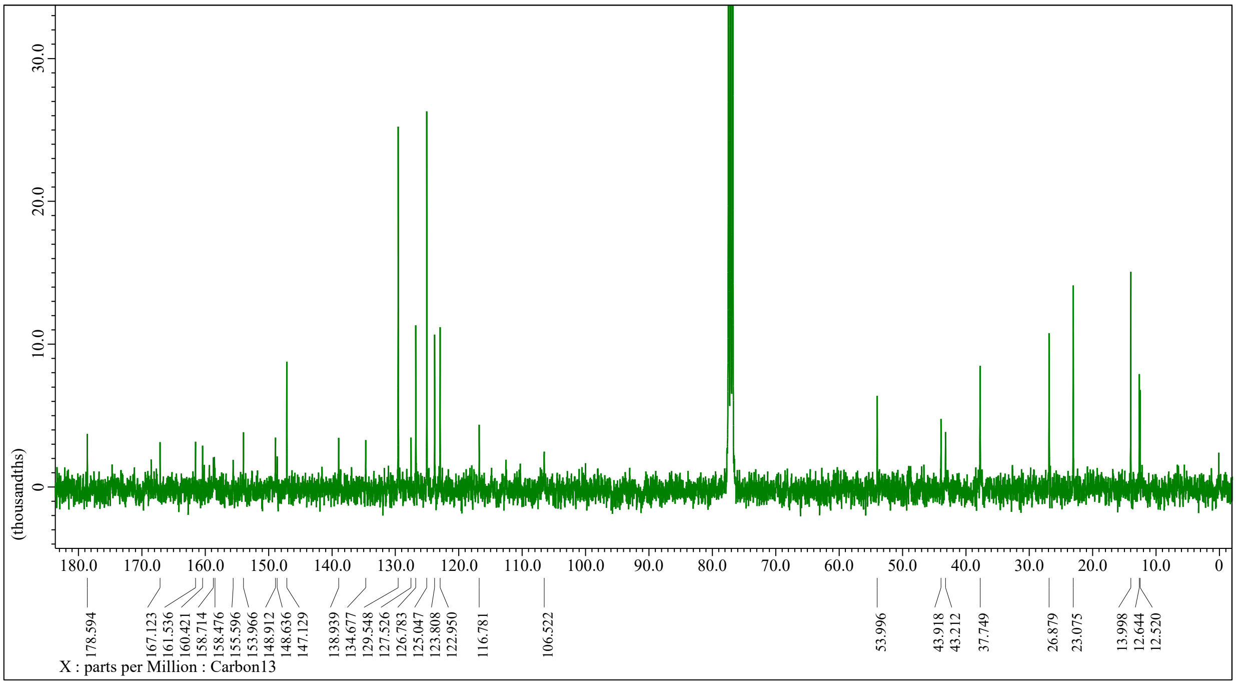

Figure S5. ^{1}H (upper, 400 MHz, CDCl$_3$) and ^{13}C (lower, 101 MHz, CDCl$_3$) NMR spectra of **2-CBu$_2$**.

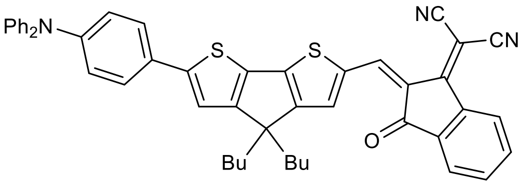

3-CBu$_2$

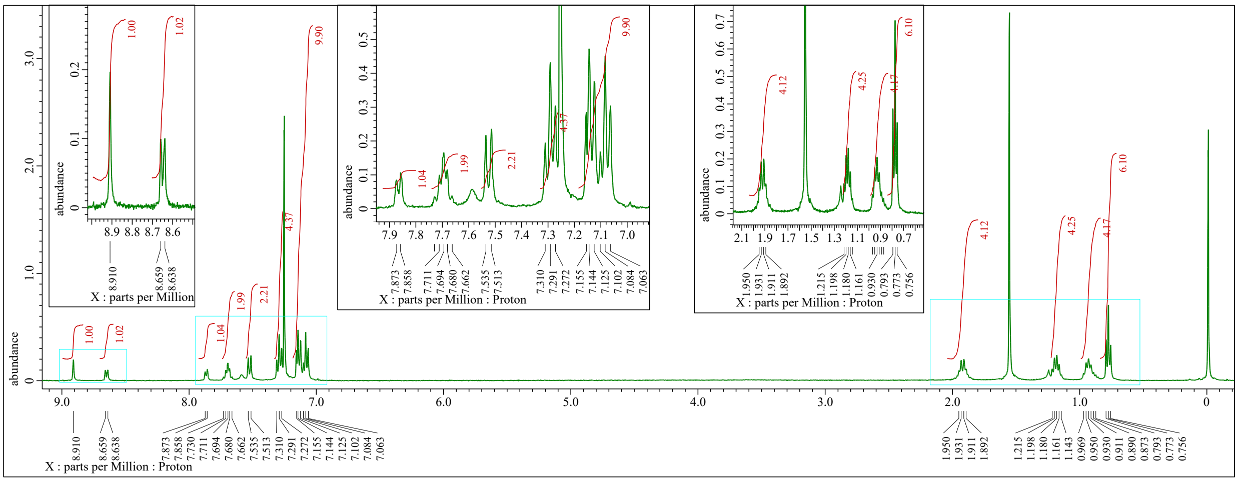

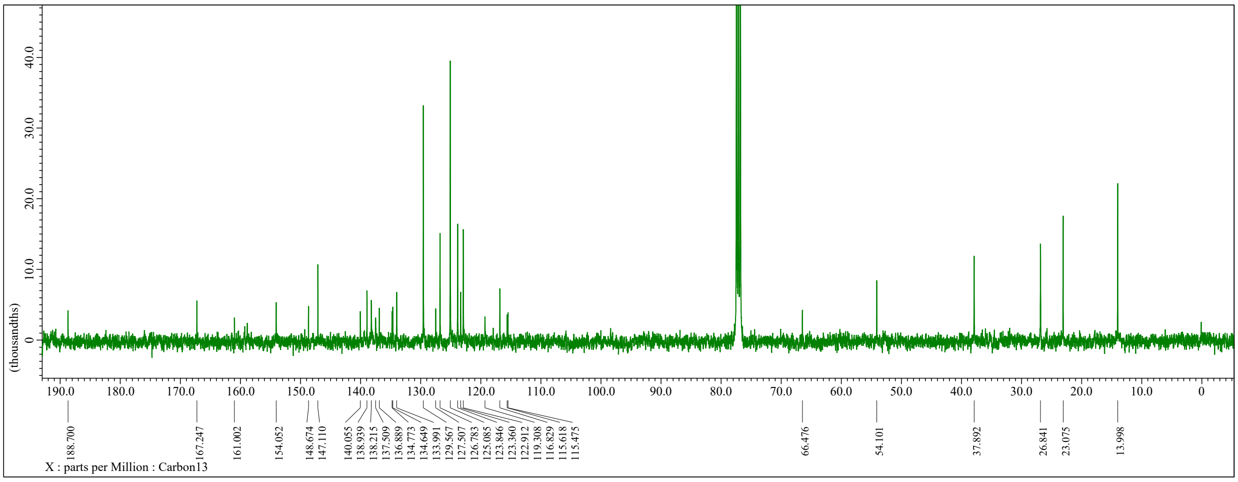

Figure S6. ^{1}H (upper, 400 MHz, $CDCl_3$) and ^{13}C (lower, 101 MHz, $CDCl_3$) NMR spectra of **3-CBu$_2$**.

3. Solvatochromic Properties

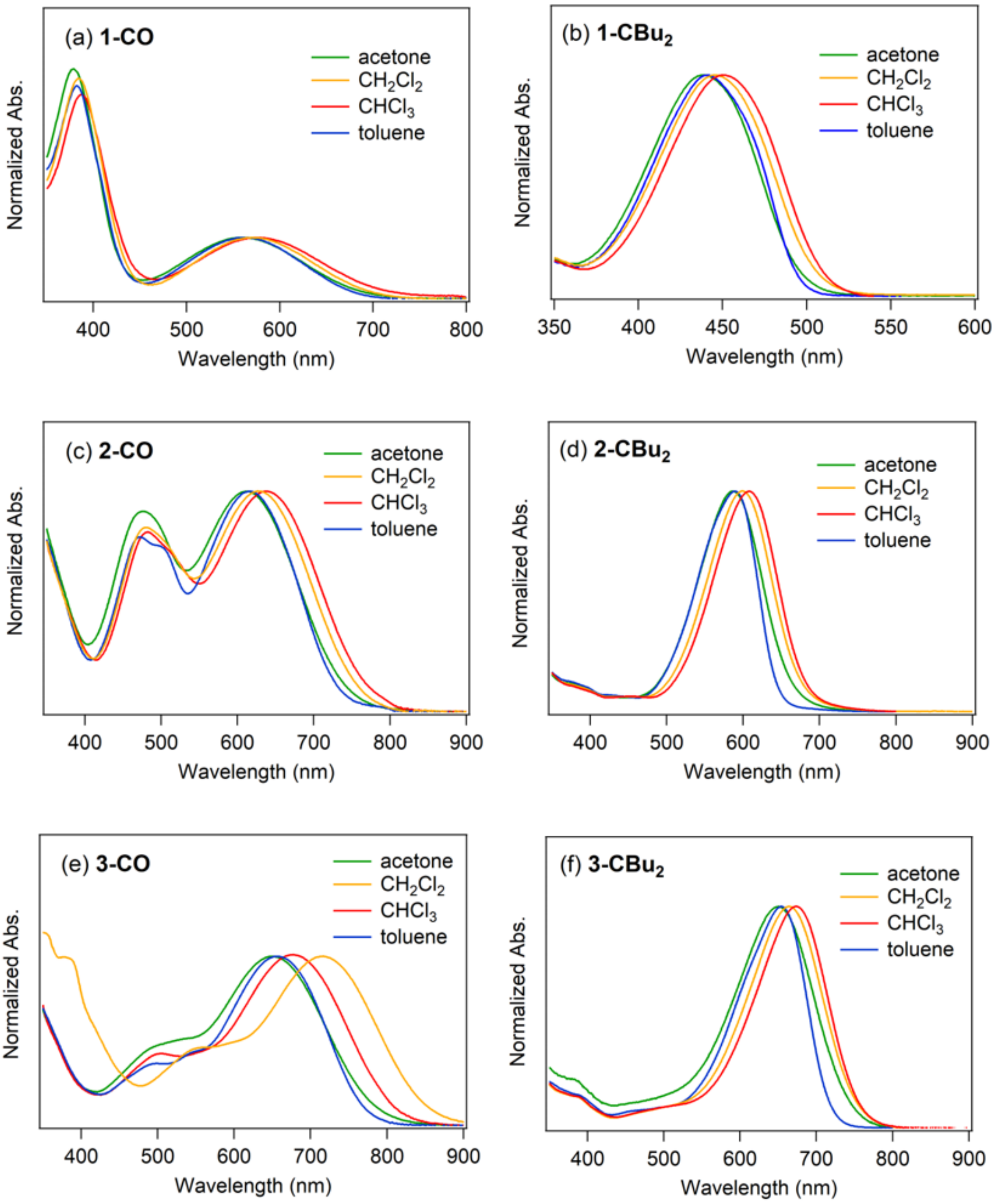

Figure S7. UV-vis-NIR spectra of (a) **1-CO**, (b) **1-CBu$_2$**, (c) **2-CO**, (d) **2-CBu$_2$**, (e) **3-CO**, and (f) **3-CBu$_2$** in various solvents (acetone, dichloromethane, chloroform, and toluene) at room temperature, normalized at the lowest-energy absorption maxima.

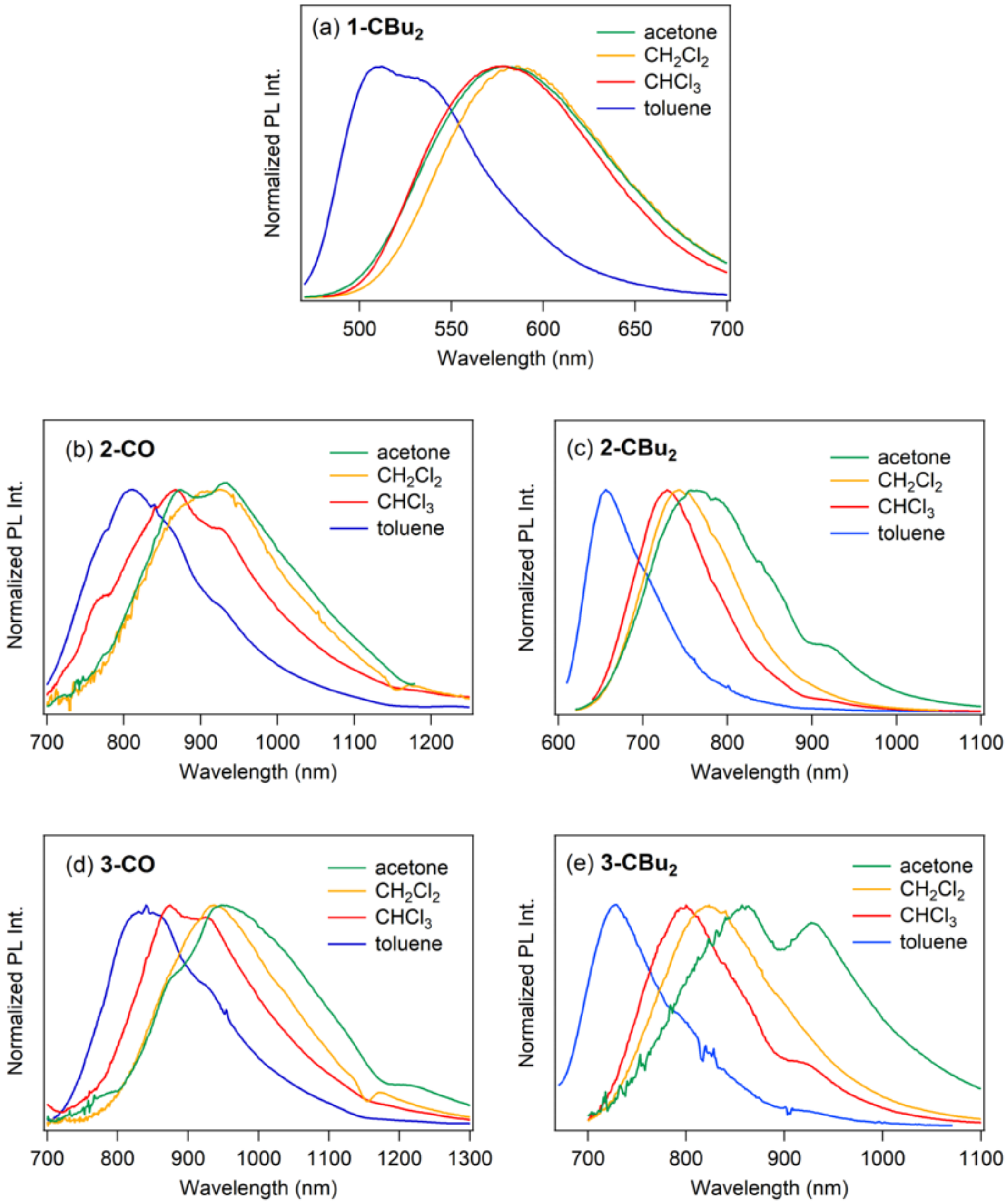

Figure S8. Photoluminescence spectra of (a) **1-CBu2**, (b) **2-CO**, (c) **2-CBu2**, (d) **3-CO**, and (e) **3-CBu2** in various solvents (acetone, dichloromethane, chloroform, and toluene) at room temperature, normalized at the highest-energy emission maxima.

4. Theoretical Calculations

Table S1. Calculated HOMO and LUMO energy levels ($E_{\text{HOMO, calc}}$ and $E_{\text{LUMO, calc}}$, respectively) and HOMO/LUMO gaps ($\Delta E_{\text{H–L, calc}}$) of **1-CO–3-CO** and **1-CBu2–3-CBu2** (M062X/6-31G(d,p) level of theory with the SMD solvation model in dichloromethane).

Compd	$E_{\text{HOMO, calc}}$ (eV)	$E_{\text{LUMO, calc}}$ (eV)	$\Delta E_{\text{H–L, calc}}$ (eV)
**1-CO**	−6.14	−1.93	4.21
**1-CBu2**	−6.02	−1.22	4.80
**2-CO**	−6.16	−2.37	3.79
**2-CBu2**	−6.05	−2.12	3.93
**3-CO**	−6.15	−2.49	3.66
**3-CBu2**	−6.01	−2.34	3.67

Table S2. Contributions of donor, π-spacer, and acceptor units to molecular orbitals in **1-CO–3-CO** and **1-CBu2–3-CBu2** obtained by the method in the literature (Lu, T.; Chen, F. Multiwfn: A Multifunctional Wavefunction Analyzer. *J. Comput. Chem.*, **2012**, *33*, 580–592).

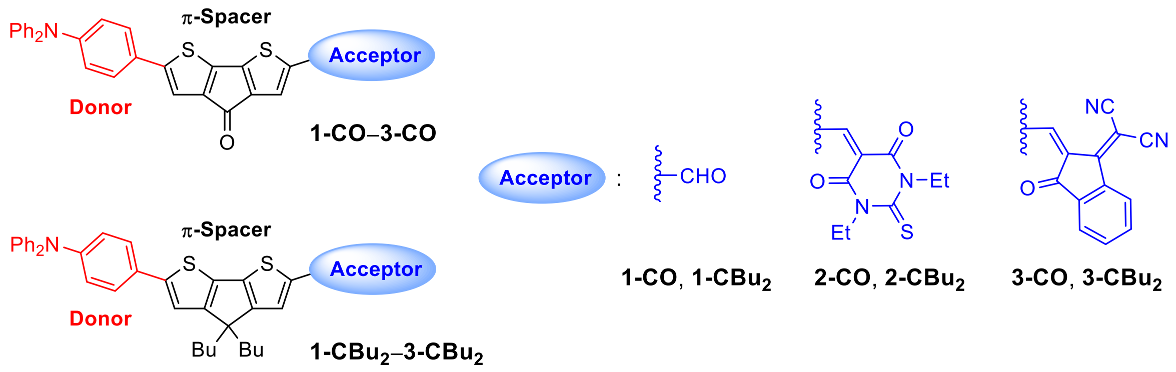

Table S2-1. Contributions of donor, π-spacer, and acceptor units to molecular orbitals in **1-CO** and **1-CBu2**.

MO	1-CO			1-CBu2		
	Donor	π-Spacer	Acceptor	Donor	π-Spacer	Acceptor
**LUMO+1**	5.2	66.6	28.2	47.8	38.0	14.2
**LUMO**	4.0	89.6	6.4	11.1	68.8	20.1
**HOMO**	80.5	18.7	0.8	68.5	29.8	1.7
**HOMO–1**	32.7	63.3	4.0	40.3	55.7	4.0

Table S2-2. Contributions of donor, π-spacer, and acceptor units to molecular orbitals in **2-CO** and **2-CBu$_2$**.

MO	2-CO			2-CBu$_2$		
	Donor	π-Spacer	Acceptor	Donor	π-Spacer	Acceptor
LUMO+1	0.6	67.2	32.2	25.5	45.2	29.3
LUMO	3.5	59.4	37.1	4.4	45.0	50.6
HOMO	77.9	19.0	3.1	65.9	28.0	6.1
HOMO–1	28.8	51.4	19.8	38.8	43.7	17.5

Table S2-3. Contributions of donor, π-spacer, and acceptor units to molecular orbitals in **3-CO** and **3-CBu$_2$**.

MO	3-CO			3-CBu$_2$		
	Donor	π-Spacer	Acceptor	Donor	π-Spacer	Acceptor
LUMO+1	1.3	47.7	51.0	1.8	12.1	86.1
LUMO	2.2	40.9	56.9	2.8	32.3	64.9
HOMO	76.0	20.3	3.7	60.8	31.2	8.0
HOMO–1	28.8	50.8	20.4	41.9	39.4	18.7
